# Gender, age and socioeconomic variation in 24-hour physical activity by wrist-worn accelerometers: the FinHealth 2017 Survey

**DOI:** 10.1038/s41598-019-43007-x

**Published:** 2019-04-25

**Authors:** Heini Wennman, Arto Pietilä, Harri Rissanen, Heli Valkeinen, Timo Partonen, Tomi Mäki-Opas, Katja Borodulin

**Affiliations:** 1 Public Health Evaluation and Projection Unit, National Institute for Health and Welfare, Helsinki, Finland; 2 Ageing, Disability and Functioning Unit, National Institute for Health and Welfare, Helsinki, Finland; 3 Mental Health Unit, National Institute for Health and Welfare, Helsinki, Finland; 4 Department of Social Sciences, University of Eastern Finland (UEF), Kuopio, Finland

**Keywords:** Public health, Epidemiology, Risk factors

## Abstract

Assessing movement over 24 hours increases our understanding of the total physical activity level and its patterns. In the FinHealth 2017 Survey, a population-based health examination study, 940 participants between 25 and 93 years were instructed to wear an accelerometer (Actigraph GT9X Link) on their non-dominant wrist for 24 hours on 7 consecutive days. Physical activity information was extracted from 100-Hz triaxial 60-second epoch data as average vector magnitude counts per minute (VM cpm). Results were analyzed by gender, 10-year age-groups, employment status, and education. Hourly means were plotted and compared. Analyses included 915 participants (44% men) who wore the device at least 10 hours on 4 or more days, with mean wear time being 149.5 hours (standard deviation of 615.2 minutes).Women had higher average VM cpm than men (p < 0.001), with significant gender differences in all age-groups until 65 years and older. Total physical activity was lower with age, unemployment, and retirement, where the hourly patterns mirrored the findings. Our findings agree with previous large-scale wrist-accelerometry data, but extend current knowledge by providing data on gender and socioeconomic variation in physical activity across 24 hours in a population-based adult sample representing a broad age range.

## Introduction

Physical activity can be considered as the accumulation of movement throughout the day, independent of type, location or purpose^1^. Technical development of accelerometers has facilitated an assessment of people’s 24 hour movement pattern and physical activity level during everyday life, also in large-scale population cohorts. Wrist-worn accelerometers have become increasingly popular for assessment of physical activity over 24 hours since they have shown good wear compliance^2,3^. Wrist-accelerometry has long been used within sleep research to estimate sleep duration, sleep efficiency, and the circadian rest-activity rhythm, often in clinical samples^4^. Many large-scale studies with adult populations have now incorporated wrist-accelerometry in their protocols, such as for example the UK Biobank^2^, the Whitehall II Study^5^, the NHANES 2011-2012^3^, the Rotterdam Study^6^, and Pelotas birth cohorts^7^.

Only few studies have yet described the 24-hour physical activity levels in a general adult population. Results from the UK Biobank study^2^ are among the most extensive so far; showing hourly, daily and seasonal variation of activity among adults (aged 45 to 79 years). In Finland, Husu *et al*.^8^ examined daytime physical activity and the times spent in different intensities of physical activity among adults (aged 18 to 85 years) using hip-worn accelerometers. According to the results, men were more sedentary than women, and younger adults had more moderate-to-high intensity physical activity compared to older ones^8^. Wrist-worn accelerometers produce substantially higher output as compared to hip-worn or waist-worn accelerometers^9–11^. To date, there is yet no consensus on the best method or criteria for detecting the type or the intensity of physical activity from the wrist-worn accelerometer data^10,12^. Hip-worn and wrist-worn accelerometer outputs correlate positively, and wrist-worn accelerometers have proven feasible in assessing total physical activity level^9,11,13^. In general, very simple summary measures such as total, median or mean activity counts or activity related acceleration per day, can be used to describe the overall physical activity level^2,10,14,15^. Furthermore, describing the accumulation of physical activity and the daily physical activity pattern over 24 hours enable visualization of data and better understanding of the amount of activity performed by more sedentary subgroups; minimizing a potential loss of information related with cut-points^16^.

To contribute to the current gap of knowledge to describe and define 24-hour physical activity and wrist-accelerometry at the general adult population, our aim is to examine the total physical activity levels and hourly activity patterns assessed by wrist-worn accelerometers among adults in Finland. We aim to study the variation by gender, age, employment and education.

## Material and Methods

**Figure 1:**
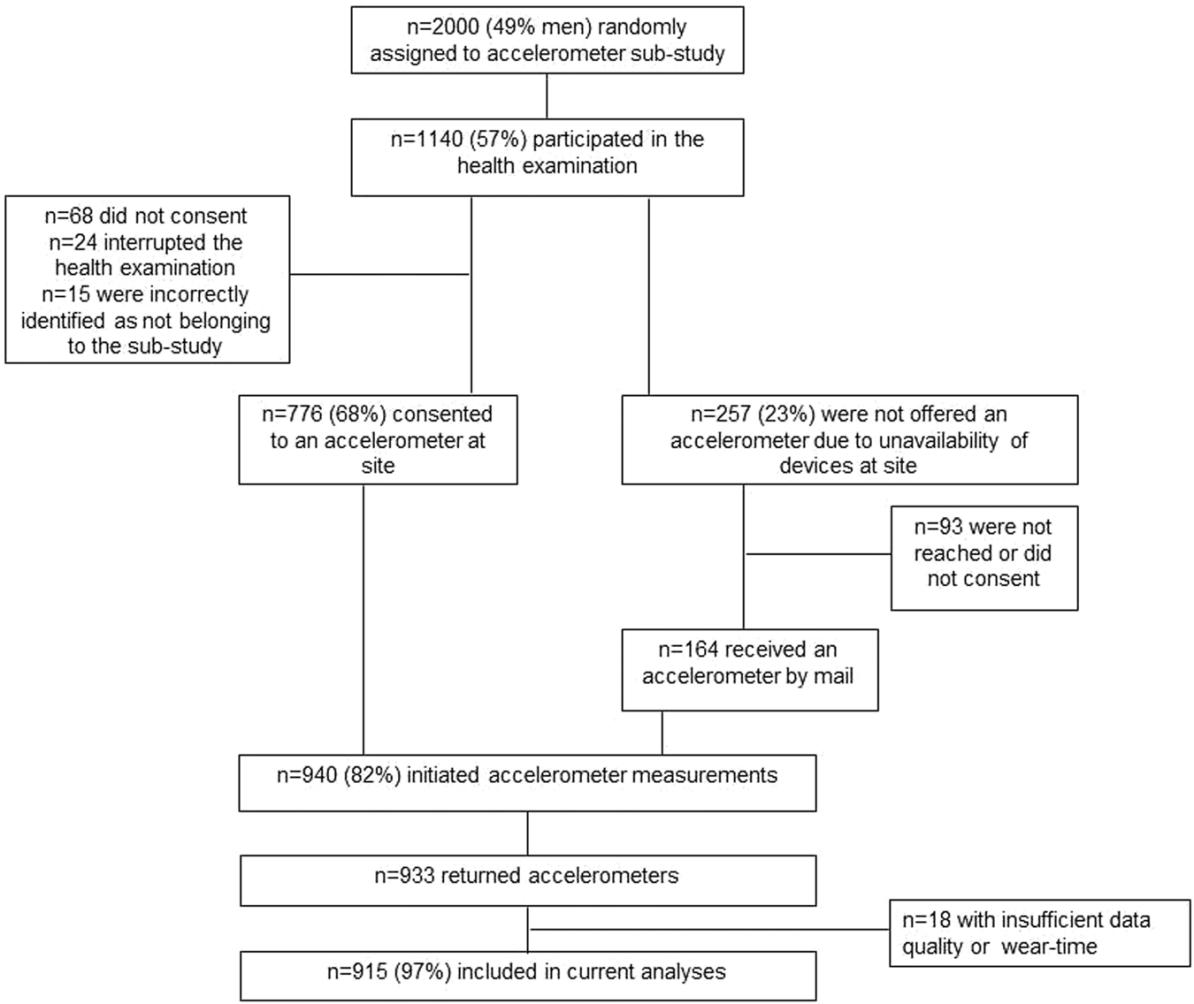
Flow chart of participation in the accelerometer sub-study.

Data comprise the FinHealth 2017 Survey, a population-based health examination study conducted in 2017. The FinHealth 2017 Survey protocol is described in detail elsewhere^17^. Briefly, 10000 adults over 18-years of age living in Finland were invited to participate by two-stage cluster sampling from the population register. The study included questionnaires and a health examination where measurements on body composition, blood pressure, functional capacity and blood sampling were undertaken. Out of the initial cohort, 2000 persons aged 25 or more were randomized into a “Physical activity and sleep” sub-study. Totally 1140 subjects (57%) of the sub-study participated in the health-examination where accelerometers were offered to participants as a part of the measurements. The participation into the device-based measurements of physical activity is described in a flow chart below (Fig. 1). The FinHealth 2017 Survey was approved by the Coordinating Ethics Committee for the Helsinki-Uusimaa hospital district. A written informed consent was obtained from all participants. All methods were performed in accordance with the relevant guidelines and regulations.

### Accelerometer measurements

At the health examination site, participants belonging to the “Physical activity and sleep” sub-study were given a triaxial wrist-worn accelerometer (Actigraph GT9X Link, Actigraphcorp, Pensacola, USA) and advised on the use of it by trained nurses. Accelerometers were initialized to collect acceleration data on three axes at 100 Hz sampling rate. The accelerometers were worn on the non-dominant wrist for 7 days, beginning from the visit at the health examination. Accelerometers were instructed to be removed only if entering sauna or swimming. Participants were asked to keep a diary to mark their bedtimes and possible non-wear periods. On the 7^th^ morning of measurements subjects could take the accelerometer off and they were told to return the device and the diary to the National Institute for Health and Welfare by prepaid mail.

Subjects who visited the health examination but for whom there were no accelerometers available at site (n = 257), were contacted afterwards by phone and offered an accelerometer. Those who agreed to take a device were mailed an accelerometer and a diary, together with written instructions for the measurements. These subjects were instructed to start wearing the device when receiving it and to end the measurements at the 7^th^ morning and return the device and the diary to the National Institute for Health and Welfare by prepaid mail.

Totally 776 persons were given an accelerometer in person at the health examination site and 164 persons received an accelerometer together with written instructions by mail (Fig. 1). Out of 940 initialized accelerometer measurements, 933 devices were returned. The diary was returned by 918 participants.

### Accelerometer data processing

Accelerometer data processing was done using the Actilife software version 6.13.3 (Actigraphcorp, Pensacola, USA) using vector magnitude (VM) counts in 60 second epoch data, i.e., counts per minute (cpm). The VM is calculated as the square root of the sum of the squared counts in three (x, y, z) axis.

As a first step, the Cole-Kripke algorithm was applied to score every 60 s epoch as asleep or awake, and the modified Tudor-Locke algorithm was applied to identify sleep periods^18–20^. Briefly, the Tudor-Locke algorithm makes use of the result by the Cole-Kripke algorithm and it determines the in-bedtime where first 5 consecutive minutes asleep are identified. Thereafter the algorithm identifies wake time as the time point where 10 consecutive minutes of awake are observed in the data following a bedtime. The minimum time between bedtime and wake time must be at least 160 minutes and the maximum length of the sleep period cannot exceed 1440 minutes.

After automatic identification of sleep periods the data were checked against the diary information for the number of sleep periods. If data included more sleep periods as compared to the diary and if the sleep periods were separated by less than 4 hours, the periods were either united or the extra sleep periods were removed depending on the notes in the diary. Furthermore, sleep data were also checked against self-reported non-wear times for potential overlapping in case of which the false sleep-period was removed. Sleep times per se were not altered.

After the detection of sleep periods the data were analyzed for non-wear by applying the Choi *et al*. algorithm to the VM data^21,22^. The algorithm checks the data for 90 consecutive minutes of zero VM counts, allowing for up to 2 minutes of non-zero counts, using a floating 30 minute window. For this non-wear checking process sleep-periods were marked as wear-time to avoid misclassification as non-wear. Finally, the 60 s epoch data files without non-wear periods were exported as VM cpm into csv-format for further analyses in external statistical software. Participants with minimum of 4 days including 10 hours of wear time from midnight-to-midnight were included in current analyses (n = 915).

### Sociodemographic factors

Information about employment status and education were assessed on a health questionnaire. Employment status was grouped into working (full-time or part-time), unemployed, retired, student, and other (including family leave and stay-at-home mothers/fathers). Education was defined as low, mid or high, according to completed educational years standardized by birth cohort. To explore levels and hourly patterns of physical activity the participants were divided into strata by gender and 10-year age-groups (25–34; 35– 44; 45–54; 55–64; 65–74; and 75+). Hourly patterns were also studied by weekday.

### Statistical methods

Differences in basic characteristics and the average VM cpm between genders were studied by t-test and between age-, employment- and education groups by analysis of variance (ANOVA). The employment- and education group comparisons were adjusted for age and gender. The average VM cpm were plotted by hour over 24-hours and the means between two consecutive hours by gender and age-group were compared by Wilcoxon signed-rank test for related samples with Bonferroni correction for multiple tests. All statistical analyses were conducted using SAS 9.3 (SAS Institute Inc, Cary, NC).

## Results

Those who returned the accelerometer (n = 933) were more often women (56% vs. 47%) and not as often aged 25–34 (13% vs. 18%) or above 85-years (3% vs. 10%), compared to the rest of the sub-study sample (n = 1067, including those who did not attend the health examination, those who did not receive an accelerometer and those who did not return the device). Descriptive characteristics, the mean wear time in minutes and number of valid days for included participants are shown by gender in Table 1. Women accumulated higher average VM cpm than men (Table 1). No statistically significant differences between genders in wear time or number of valid days was observed.

**Table 1:** Descriptive information (mean and SD, frequency [%]) about the study population.

	Men (n = 400)	Women (n = 515)	p-value
Age (years)	55.9 (16.3)	55.5 (16.6)	0.711
BMI (kg/m^2^)	27.6 (4.2)	27.3 (5.6)	0.350
Height (meters)	1.77 (0.07)	1.63 (0.07)	<0.0001
Wear time (minutes)	8988.2 (636.5)	8950.8 (598.3)	0.362
Wear time (hours)	149.8	149.2	
Valid days (number)	6.6 (0.6)	6.5 (0.6)	0.139
VM cpm	1494.6 (425.4)	1736.6 (537.7)	<0.0001
Education	n = 393	n = 510	0.161
Low	27.7%	31.8%	
Mid	31.3%	33.3%	
High	41.0%	34.9%	
Employment	n = 399	n = 512	0.002
Working	53.9%	52.2%	
Retired	36.6%	34.6%	
Unemployed	6.8%	4.3%	
Student	1.8%	4.3%	
Other	1.0%	4.7%	

Gender differences in average VM cpm were significant in all age-groups except the oldest two (Table 2). Within both genders there were significant differences between age-groups in the average VM cpm, with lower values in the older age-groups of 65 −74 and 75+ years than in the three youngest age-groups.

**Table 2:** Average daily VM cpm by gender and age-group. Statistical comparison between age-groups within and between gender.

Age	Men*		Women*		p between genders
	N	Mean (SD)	N	Mean (SD)	
25–34 years	53	1655.2 (450.0)	73	1936.8 (500.5)	0.002
35–44 years	69	1641.2 (328.5)	83	1955.8 (434.8)	<0.0001
45–54 years	76	1641.9 (415.4)	103	1941.8 (469.6)	<0.0001
55–64 years	71	1481.9 (340.9)	106	1847.0 (511.3)	<0.0001
65–74 years	75	1385.4 (418.2)	72	1441.8 (365.5)	0.386
75+ years	56	1124.0 (362.0)	78	1167.1 (387.3)	0.515

* = statistically significant difference between age-groups within gender.

**Figure 2:**
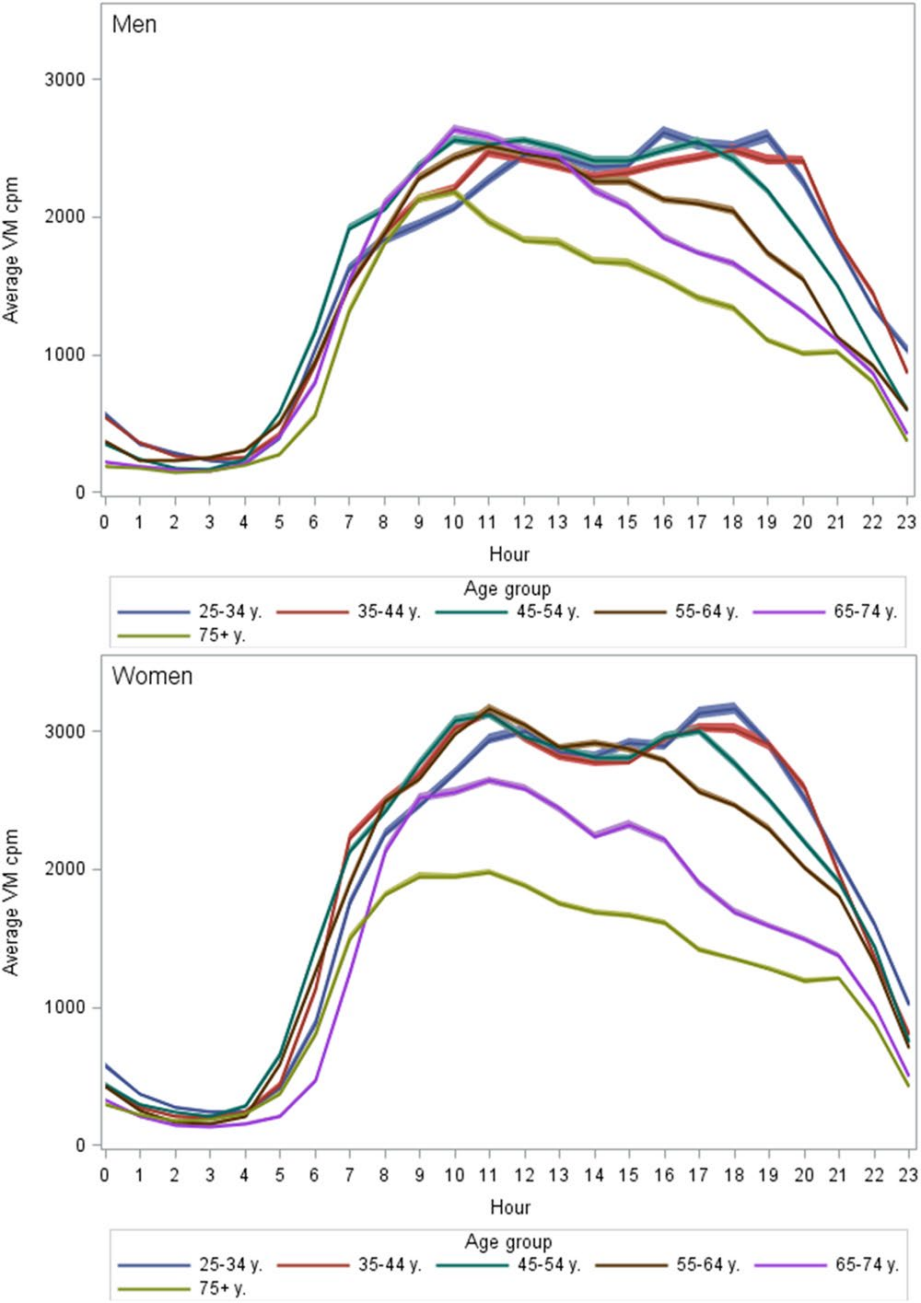
Hourly pattern of physical activity by age-group and gender. Upper figure men, lower figure women.

In Fig. 2 the variation in physical activity over 24 hours by gender and age-groups is presented. In general, a low activity level at night (between 1 a.m. and 4 a.m.), a considerable rise in activity level during morning hours (around 5 and 6 a.m.) (Supplementary Table S1), a higher activity level during daytime hours and a more or less pronounced drop in activity level towards evening hours (around 8 p.m.) were observed (Fig. 2).

Differences in average daily VM cpm by age-groups are mirrored in the different hourly patterns between age groups. In the oldest participants the VM cpm level of two consecutive hours showed few significant differences while in younger men and women several consecutive hours in the morning and in the evening differed between each other (Supplementary Table S1). In youngest women and two oldest groups in both genders, changes in activity level from one hour to another were significant from the 5^th^ and 6^th^ hour until the 8^th^ hour. In women aged 35-44 and men and women 45-64 years the rise in activity level was significant between consecutive hours from the 5^th^ and 6^th^ hour until the 9^th^ or 10^th^ hour (Supplementary Table S1). In men, the youngest age group reached their highest activity levels in the later afternoon hours (after 16^th^ hour) whereas the peak levels in other age groups occurred earlier in the day, particularly for the oldest two age-groups Also in women, the youngest reached their highest activity levels in late afternoon hours (Fig. 2 and Supplementary Table S1), whereas in the two oldest age-groups the hours with highest average VM cpm were observed before noon. In women, all ages had a peak in activity in the morning hours and in the youngest three age-groups a second peak was observed around 6 p.m. in late afternoon.

**Figure 3:**
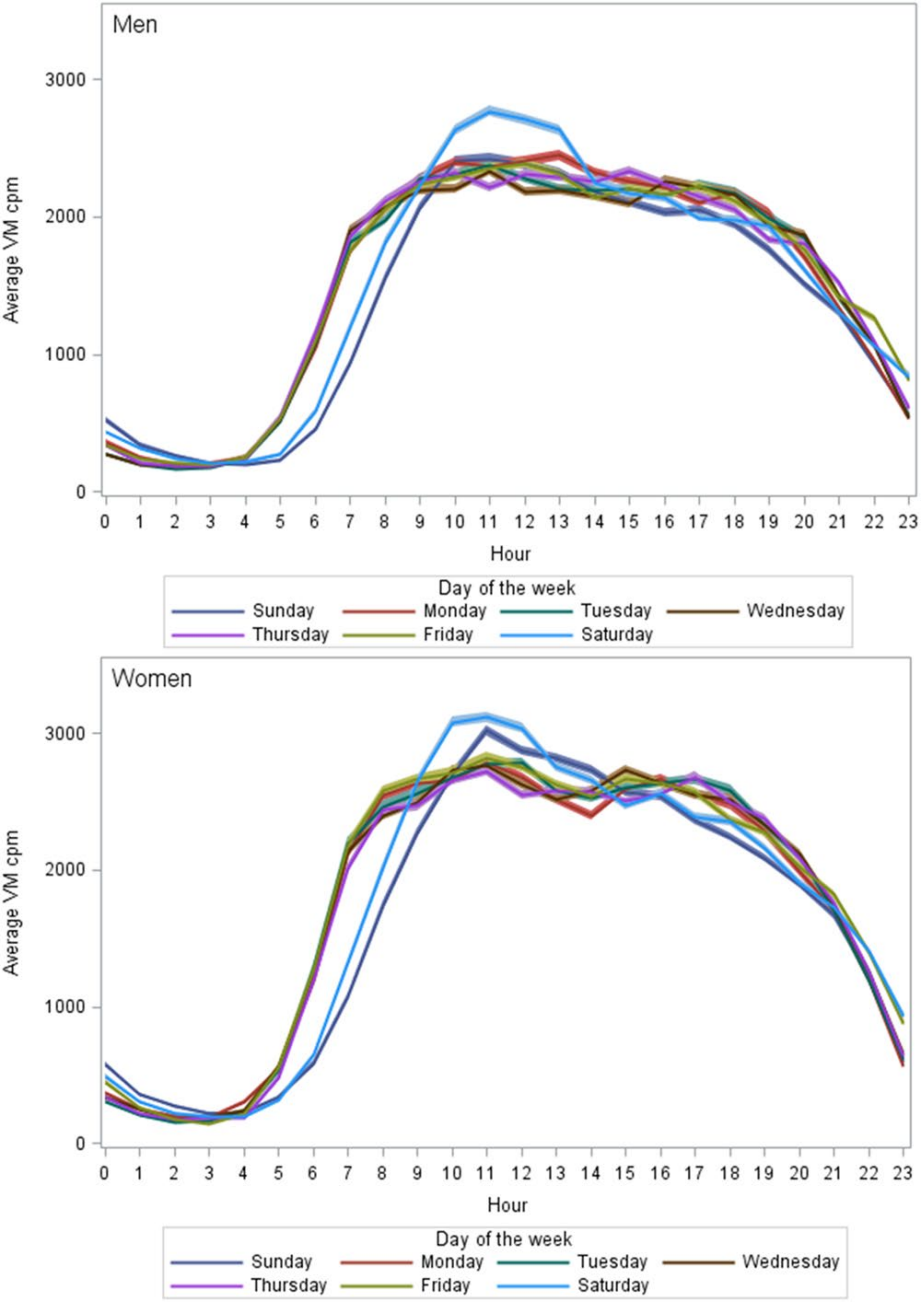
Hourly pattern of physical activity by weekday. Upper figure men, lower figure women.

The hourly patterns of activity were different between weekdays (Fig. 3). In both genders the weekend days (Saturday and Sunday) showed a different hourly pattern as compared to the other weekdays (Monday through Friday) which all were very similar to each other. In men, there was a clearly higher peak in activity level during Saturdays than on any other weekdays. Also in women, higher activity levels were observed on Saturdays, but also on Sundays. The comparisons of VM cpm between consecutive hours showed that the change in activity level from one hour to the next were mostly significant only in early morning and late evening hours on all days of the week. For both genders, the activity level on weekend mornings before 9 a.m. was clearly lower as compared to the rest of the weekdays (Fig. 3 and Supplementary Table S2).

**Figure 4:**
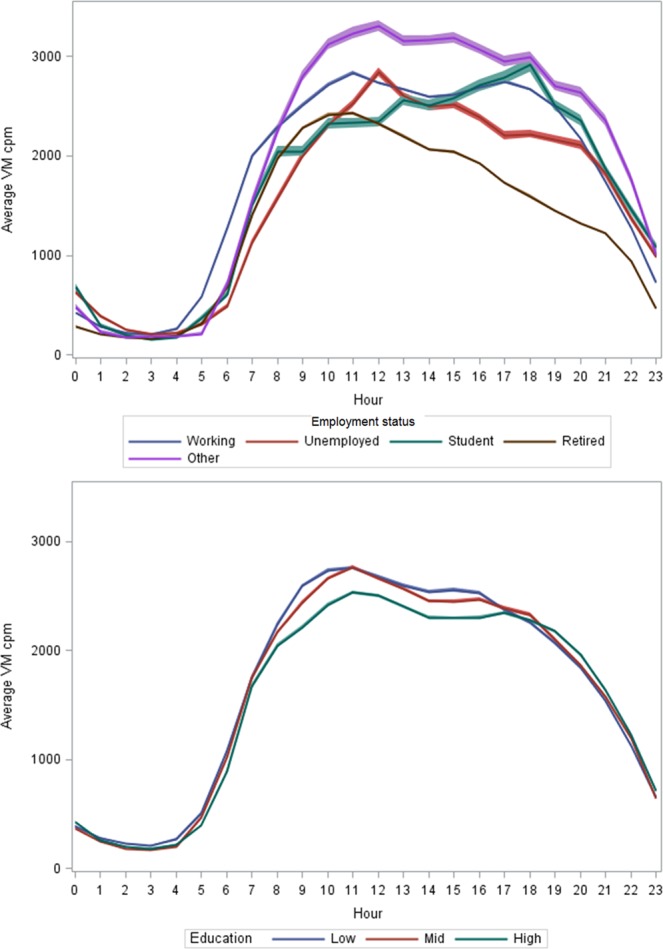
Hourly patterns of physical activity by employment status (upper figure) and education (lower figure).

Significant differences in physical activity levels were also observed for employment status, but not educational level (Table 3). Students and the retired had the lowest average VM cpm, when adjusted for age and gender. The group of others showed the highest average VM cpm of all employment groups. The retired and the unemployed showed one clear peak in activity level in the morning hours, followed by a tapering towards evening hours (Fig. 4). The employed sustained their activity level throughout daytime whereas students had the slowest rise in activity level during morning hours with the peak in activity occurring in the early evening.

**Table 3:** Mean VM cpm and standard error (SE) by employment status and educational level.

	Mean (SE) VM cpm*	
Employment status (missing n = 4)		p < 0.0001
Student n = 29	1446.6 (87.0)	
Retired n = 323	1477.7 (34.6)	
Unemployed n = 49	1533.3 (62.8)	
Working n = 482	1751.1 (23.6)	
Other n = 28	1836.6 (83.8)	
Education level (missing n = 12)		p = 0.113
Low n = 271	1651.6 (27.0)	
Middle n = 293	1632.2 (25.9)	
High n = 339	1579.8 (24.0)	

*Adjusted for age and gender.

## Discussion

We observed that women had higher average VM cpm than men in all except the oldest two age-groups. An inverse association between age and average VM cpm was observed within genders. The hourly patterns of activity were different between age-groups and weekdays, as well as employment status. The oldest had the lowest level of activity throughout the day and the hourly pattern in students was opposite to other employment groups with activity level rising towards evening hours. Furthermore, in both men and women, the hourly activity patterns on Saturday and Sunday were different from the other weekdays.

Even if only few results regarding physical activity based on 24-hour accelerometer-based data are available from large population-based cohorts, the gender differences in activity level have already been observed by others as well. In one of the largest studies applying wrist-accelerometry, the UK Biobank study, women had higher mean acceleration values as compared to men, except in those aged 45 to 54-years^2^. Men had lower physical activity level that tended to decline more by age, as compared to women. In three different Brazilian birth cohorts, women gender and low socioeconomic status where associated with higher physical activity as measured by wrist-worn accelerometers^7^. However, similar to our finding, there are some other studies that have applied wrist-worn devices in adults aged 60 years or older and not observed any differences between genders^5,23^.

Based on questionnaire data, women engage more in household activities than men^24^, which could be a reason for differences in the observed wrist-accelerometer output between genders. According to Shiroma *et al*.^10^ the hip-worn and the wrist-worn monitors may be engaged in somewhat different behaviors during the course of the day. Where the hip-worn devices may assess the whole-body movements, the wrist-worn devices measure most of the whole-body movements in addition to the movements being specific to wrist- or upper body. Furthermore, a wrist-worn accelerometer reaches 25% of total daily activity earlier than a hip-worn device, but a hip-worn device reaches 75% faster than the wrist^10^. This indicates that towards evening hours the movement type might become different and include less ambulatory, whole-body movements resulting in less movement to be captured for a hip-worn accelerometer^10^. More exact reasons for the differences in the accelerometer output between genders for different wear locations remain to be studied further, in order to improve the understanding of gender differences in physical activity.

Our result also shows that older adults had significantly lower VM cpm than younger ones. Other large-scale studies have consistently shown a decline in device-based total physical activity with increasing age^2,7,15,16,25,26^. Doherty *et al*.^2^ reported total physical activity level over 24-hours to differ strongly between age-groups in both genders, with approximately 7.5% lower mean physical activity level per decade of age. Also in Brazilian children, adolescents and young adults, an inverse linear association was observed between age and overall physical activity^7^ and in Dutch adults older age was associated with a more stable hourly pattern between days, when examined by wrist-accelerometry^6^.

We present the hourly patterns of activity in adults of a broad range of ages. We only compared the activity level for every two consecutive hours, but it was observed that the youngest participants aged 25- to 34 years, reached their highest average VM cpm in late afternoon or evening hours. Further, particularly for women in the mid-age, the rise in activity level continued significant until later hours in the morning. In the oldest men and women there were only few significant differences in activity level between consecutive hours, which to some extent confirms that there was less variation in activity level during 24 hours in the oldest participants. Our observations are supported by some previous studies that have shown the activity level in older persons to peak early in the day (around 10 a.m.) and a prolonged taper throughout the rest of the day^10,27^. The differences in physical activity between age-groups have also been most prominent in the afternoon hours^2^, older participants showing a decline in activity level after a peak in the morning, whereas younger subjects sustain their activity level until later hours in the afternoon^16^.

One important underlying factor for the hourly patterning of activity is chronotype. The chronotype of adolescents^28^ and young adults^29^ is more often evening type, and chronotype may change with ageing towards more of morning type^30^. However, it may be that the morning types become more frequent among older age-groups due to an increase in premature deaths among the evening types^30,31^. Age-related differences in the hourly pattern of physical activity can also be a result of household or family size, where especially the presence of younger children in the household could result in a more active afternoon and evening time as compared to a person living as single.

As expected, the hourly patterns of activity differed between weekdays and there was a higher, albeit later peak in activity level on weekend days, especially Saturdays, in both genders. It is known that physical activity during weekend days may be different from other weekdays, but also that Saturdays and Sundays may differ^2,32^. Especially women may show slightly higher physical activity levels on Saturdays compared to the average weekday^33^. Older persons show a more stable pattern between weekdays^6^ and younger persons have more activity during weekend days^2,34^. Furthermore, in the current study the activity levels on weekend mornings were lower than on other weekdays and this also support the findings that people may alter their sleep times, particularly their wake-up time during free days as compared to working days^29^. Even if our result does not permit to conclude on what type of activity the peak during Saturdays include, together with previous findings the observation supports the importance of weekend days as contributors to the weekly physical activity amount in adults.

The unemployed differed in activity level and pattern from the working aged in general, indicating that not having a work to attend decrease the activity level throughout the day. Employment, but not type of employment, is associated with more activity as measured by waist-worn accelerometer, with strongest effect size in younger subjects^16^. Employed and highly educated persons seem to concentrate their physical activity on weekend days^34^. Interestingly, we observed students to have the lowest average VM cpm and the most different hourly activity pattern with a slow rise in activity level and the latest timing of peak in activity. This indicates that students may engage in more stationary activities and follow a different diurnal rhythm compared to other employment groups. However, the number of students in the sample is small and is limited to those aged 25 and older, thus limiting broad generalization of the result.

### Limitations

The potential impact of non-wear and sleeping time on the activity output needs to be acknowledged^35^. In the current study, once sleep periods were determined, they were set as wear-time in the non-wear analysis, to avoid the misclassification of sleep periods as non-wear. The separation of non-wear from actual sleep in such 24 hour data is important in order not to exclude true zeros, i.e., zero VM cpm that can occur in sleep from the analyses. In protocols for hip-worn accelerometers false non-wear identification most likely affect estimates of sedentary time. We did not assess reasons for non-wear, but due to the 24-hour protocol it is likely that longer non-wear periods occur for example due to jobs where a wrist-worn device cannot be worn (such as surgeon, nurse, chef, etc.). Thus it can be speculated that long non-wear periods in this dataset rather include activity than sedentary time, which in turn would increase rather than decrease the activity level of some sub-groups.

We decided, as many before us, to report counts, not raw acceleration. Reporting physical activity by accelerometers “counts” is popular^36^, yet their use is criticized. Counts constitute a summary measure based on the frequency and intensity of post-filtered acceleration values from the accelerometer (Actigraph LLC). Counts also can vary between accelerometer brands which makes comparability between studies difficult. Our goal was to report the total volume and hourly patterning of overall physical activity, and in this matter we believe that both counts and raw acceleration provide the same story. This is also supported by comparisons between hip-worn and wrist-worn devices that include both counts and raw acceleration^9–11^ as well as by the similarity of our findings to studies that have reported the average raw acceleration^2^.

To classify physical activity into different intensity categories, a variety of different cut points are available, but mainly when the accelerometer is worn on the waist or hip^13^. The use of cut-points to determine physical activity intensities is questionable, since cut-points are sensitive to the population and wear-site for which they were created^3^. Furthermore, for 24 hour measurements it is important to notice that a lot of the activity is also happening from non-ambulatory activities, but that many calibration studies have only used ambulatory activities when developing their prediction models^37^. Implementing machine learning techniques taught on free-living data show promising results also for wrist-worn devices to identify between activity intensities and types^38^. Once better consensus on the processing of wrist-accelerometer data are achieved it will without doubt be interesting to deepen the findings also from the current study by more specific information about intensity and type of activity throughout the 24 hours.

There is some selection in the participation to the current measurements, as typically for health examination studies where women and older persons are more likely to participate^39^. Subjects who returned the accelerometer were more likely women and in mid-age as compared to the initial sub-study sample, limiting generalizability of the result to some extent.

Strengths of this study include a fairly large, population-based and randomly selected cohort of adults representing a broad range of ages. The wrist-worn accelerometer resulted in excellent compliance and provides valuable information about the full 24 hour movement continuum. There is great value in activity pattern analyses as they provide a means to examine differences in activity volume between different subgroups of people that would not necessarily be identified using time in intensity level approaches^10^. Furthermore, the accelerometer that was used in the FinHealth 2017 Survey is one of the most widely used research monitor devices and thus enables future comparison with many existing and forthcoming studies nationally and internationally.

## Conclusions

This study is the first to report on device-based physical activity levels and hourly patterns across 24 hours in the general adult population in Finland. Findings indicate higher total physical activity in women than men in age-groups younger than 65 years. However, further work is required for the underlying sources of the gender and age differences observed by wrist-accelerometry to be better understood and specific activity types to be identified more accurately. Even if the wrist-accelerometer may capture also other than whole-body movements, the hourly patterns of physical activity highlight the differences in level and timing of activity between age and socioeconomic groups, as well as between weekdays in the population. The information is useful to consider for more targeted physical activity promotion with respect to different population groups.

## Supplementary information

Supplementary tables S1 and S2

## Data Availability

Access to FinHealth data is normally granted for researchers who are affiliated with a known institution, to study topics in line with the given ethical guidelines and Finnish legislation. The Executive Board of the FinHealth Survey approves the data access based on written requests and study plans, after which a signed data use contract between the receiving institute and the Finnish National Institute for Health and Welfare is required.
