# Supplementary material for: Gender, age and socioeconomic variation in 24-hour physical activity by wrist-worn accelerometers: the FinHealth 2017 Survey
Source: Sci Rep. 2019 Apr 25;9:6534. doi: 10.1038/s41598-019-43007-x (PMC6483989; doi:10.1038/s41598-019-43007-x)
Supplement: Supplementary file 1 — Supplementary tables S1 and S2 [file 41598_2019_43007_MOESM1_ESM.pdf]

Title: Gender, age and socioeconomic variation in 24-hour physical activity by wrist-worn accelerometers: the FinHealth 2017 Survey

Authors: Heini Wennman[1], Arto Pietilä [1], Harri Rissanen [1], Heli Valkeinen [2], Timo Partonen [3], Tomi Mäki-Opas [4], and Katja Borodulin [1]

1. Public Health Evaluation and Projection Unit, National Institute for Health and Welfare, Helsinki, Finland
2. Ageing, Disability and Functioning Unit, National Institute for Health and Welfare, Helsinki, Finland
3. Mental Health Unit, National Institute for Health and Welfare, Helsinki, Finland
4. Department of Social Sciences, University of Eastern Finland (UEF), Kuopio, Finland

Corresponding author: Heini Wennman, e-mail: heini.wennman@thl.fi

Supplementary table S1. The average VM cpm by hour for men and women by age group. The Bonferroni adjusted p-value for a significant difference when compared to the next hour is presented and highlighted in yellow color.

Hours Men	0-1	1-2	2-3	3-4	4-5	5-6	6-7	7-8	8-9	9-10	10-11	11-12	12-13	13-14	14-15	15-16	16-17	17-18	18-19	19-20	20-21	21-22	22-23	23-24
25-34	556	343	283	228	205	395	1041	1646	1875	1975	2089	2273	2454	2448	2366	2377	2611	2544	2515	2605	2283	1822	1361	1034
	0.000				0.016	<0.0001	<0.0001														0.010	<0.0001	0.009	<0.0001
35-44	532	344	254	225	239	415	924	1501	1892	2142	2211	2462	2416	2366	2294	2327	2387	2431	2501	2417	2409	1840	1455	848
	<0.0001	0.020				<0.0001	<0.0001														<0.0001	0.004	<0.0001	<0.0001
45-54	349	241	171	163	246	576	1164	1924	2066	2383	2574	2544	2553	2504	2411	2420	2469	2516	2414	2176	1846	1515	1019	587
	0.005				<0.0001	<0.0001	<0.0001		0.031											0.003	0.005	<0.0001	<0.0001	<0.0001
55-64	372	228	231	249	300	499	942	1507	1869	2269	2435	2526	2466	2429	2254	2253	2128	2097	2040	1727	1544	1122	920	601
	0.038				0.019	<0.0001	<0.0001	0.001	0.038										0.042		<0.0001	0.008	<0.0001	<0.0001
65-74	218	189	165	158	212	411	788	1531	2099	2336	2603	2554	2460	2419	2184	2066	1849	1740	1653	1491	1309	1098	864	422
						<0.0001	<0.0001	<0.0001															<0.0001	<0.0001
75+	187	171	147	155	194	269	557	1311	1825	2140	2169	1963	1826	1810	1684	1667	1555	1412	1340	1101	1012	1015	800	370
							<0.0001	0.002															<0.0001	0.001

Hours Women	0-1	1-2	2-3	3-4	4-5	5-6	6-7	7-8	8-9	9-10	10-11	11-12	12-13	13-14	14-15	15-16	16-17	17-18	18-19	19-20	20-21	21-22	22-23	23-24
25-34	573	362	268	242	238	416	891	1763	2265	2475	2703	2939	2993	2854	2812	2913	2907	3134	3175	2908	2519	2067	1623	1049
	0.000					<0.0001	<0.0001	0.031												0.014	0.001	0.000	<0.0001	<0.0001
35-44	421	267	199	192	241	437	1127	2240	2498	2692	3024	3139	2950	2815	2756	2766	2940	3026	3015	2915	2605	1972	1372	798
	0.041				0.034	<0.0001	<0.0001			0.003											<0.0001	<0.0001	<0.0001	<0.0001
45-54	429	291	224	199	286	653	1422	2134	2431	2782	3098	3144	2965	2878	2810	2801	2960	3003	2759	2486	2189	1910	1426	744
	0.012				<0.0001	<0.0001	<0.0001		0.018											0.023	0.007	<0.0001	<0.0001	<0.0001
55-64	422	243	167	152	208	589	1262	1909	2501	2654	2990	3157	3045	2878	2907	2865	2789	2581	2472	2306	2011	1806	1326	708
	<0.0001				<0.0001	<0.0001	<0.0001	<0.0001		0.024										0.000	0.022	<0.0001	<0.0001	<0.0001
65-74	327	205	149	133	156	203	461	1240	2145	2525	2559	2646	2588	2438	2242	2313	2211	1906	1699	1606	1503	1361	1013	505
						<0.0001	<0.0001	<0.0001														0.001	<0.0001	0.002
75+	296	219	175	187	229	378	806	1493	1811	1952	1935	1961	1875	1755	1677	1667	1616	1419	1348	1284	1189	1209	880	432
						<0.0001	<0.0001	0.005									0.016					0.003	<0.0001	

Supplementary table 21. The average VM cpm by hour for men and women by weekday. The Bonferroni adjusted p-value for a significant difference when compared to the next hour is presented and highlighted in yellow color.

Hours: MEN	0-1	1-2	2-3	3-4	4-5	5-6	6-7	7-8	8-9	9-10	10-11	11-12	12-13	13-14	14-15	15-16	16-17	17-18	18-19	19-20	20-21	21-22	22-23	23-24
Mon	362	253	197	206	253	519	1049	1752	2059	2274	2398	2370	2406	2449	2320	2259	2215	2108	2177	2025	1711	1336	950	536
					0.006	<0.0001	0.005	0.037	0.048													0.002	0.002	0.006
Tue	273	192	166	179	239	506	1078	1813	1978	2280	2312	2376	2278	2208	2189	2208	2162	2226	2173	1993	1843	1415	1071	538
						0.000	0.022														0.002	<0.0001	<0.0001	<0.0001
Wed	273	196	181	184	256	520	1092	1903	2070	2197	2202	2337	2183	2190	2153	2094	2263	2216	2165	1935	1869	1428	1062	539
						0.006	<0.0001															0.001	0.000	<0.0001
Thu	337	213	189	185	244	547	1157	1862	2117	2263	2318	2216	2312	2291	2254	2332	2238	2146	2056	1833	1805	1521	1088	605
					<0.0001	0.002	<0.0001														0.002		<0.0001	0.001
Fri	339	243	208	200	253	530	1103	1755	2060	2241	2289	2355	2388	2313	2145	2207	2159	2224	2119	1961	1762	1416	1261	816
						<0.0001	<0.0001	0.002												0.050		0.000	<0.0001	<0.0001
Sat	435	316	244	211	219	272	586	1192	1820	2229	2632	2768	2711	2631	2252	2170	2137	1993	1981	1932	1616	1314	1061	835
						<0.0001	<0.0001														0.021	0.012	<0.0001	<0.0001
Sun	519	340	258	212	200	227	452	933	1560	2066	2408	2421	2382	2323	2183	2112	2034	2048	1944	1764	1509	1306	930	557
	0.000					<0.0001	<0.0001	0.016													0.019		<0.0001	<0.0001
Hours: WOMEN	0-1	1-2	2-3	3-4	4-5	5-6	6-7	7-8	8-9	9-10	10-11	11-12	12-13	13-14	14-15	15-16	16-17	17-18	18-19	19-20	20-21	21-22	22-23	23-24
Mon	369	250	199	191	302	539	1188	2115	2546	2632	2659	2792	2676	2513	2398	2597	2665	2560	2480	2286	1992	1712	1192	566
						<0.0001	<0.0001	0.020	0.004													<0.0001	<0.0001	<0.0001
Tue	305	203	155	159	240	534	1288	2198	2467	2565	2677	2774	2783	2578	2523	2603	2639	2665	2585	2333	2095	1707	1194	613
					0.016	<0.0001	<0.0001	0.026	0.022													0.000	<0.0001	<0.0001
Wed	333	252	179	192	241	559	1239	2136	2401	2499	2735	2767	2630	2519	2579	2732	2637	2549	2520	2337	2119	1738	1255	650
	0.002				<0.0001	<0.0001	<0.0001	0.009													<0.0001	0.005	<0.0001	<0.0001
Thu	339	214	180	183	190	480	1204	2010	2439	2469	2663	2725	2553	2585	2571	2502	2565	2680	2497	2379	2081	1759	1258	635
	0.002				0.047	<0.0001	<0.0001	0.011										0.049		0.041	0.039	0.003	<0.0001	0.004
Fri	449	265	180	147	218	555	1276	2179	2580	2664	2715	2824	2752	2628	2538	2665	2632	2572	2370	2276	2026	1822	1395	880
					0.047	<0.0001	<0.0001	0.000			0.027											<0.0001	<0.0001	0.000
Sat	489	307	218	196	199	319	645	1320	2024	2643	3080	3119	3038	2752	2662	2470	2562	2390	2354	2165	1903	1735	1408	932
	0.010				0.044	<0.0001	<0.0001			0.028											<0.0001	<0.0001	<0.0001	<0.0001
Sun	577	357	272	220	224	336	586	1068	1739	2272	2702	3020	2872	2823	2734	2571	2538	2369	2242	2089	1892	1673	1229	599
	<0.0001				0.001	<0.0001	<0.0001	0.005												<0.0001		<0.0001	<0.0001	<0.0001
